# The Effect of Feeding a Total Mixed Ration with an *ad libitum* or Restricted Pelleted Starter on Growth Performance, Rumination Behavior, Blood Metabolites, and Rumen Fermentation in Weaning Holstein Dairy Calves

**DOI:** 10.3390/ani14010081

**Published:** 2023-12-25

**Authors:** Anna Antonella Spina, Vincenzo Lopreiato, Domenico Britti, Andrea Minuti, Erminio Trevisi, Bruno Tilocca, Alessia Perri, Valeria Maria Morittu

**Affiliations:** 1 Interdepartmental Services Centre of Veterinary for Human and Animal Health (CISVetSUA), University “Magna Græcia” of Catanzaro, Viale Europa, 88100 Catanzaro, Italy; britti@unicz.it (D.B.); tilocca@unicz.it (B.T.); morittu@unicz.it (V.M.M.); 2 Department of Health Science, University “Magna Graecia” of Catanzaro, Viale Europa, 88100 Catanzaro, Italy; perrialessia10@gmail.com; 3 Department of Veterinary Sciences, University of Messina, Viale Palatucci 13, 98168 Messina, Italy; vincenzo.lopreiato@unime.it; 4 Department of Animal Sciences, Food and Nutrition (DiANA), Faculty of Agriculture, Food and Environmental Sciences, Università Cattolica del Sacro Cuore, Via Emilia Parmense, 84, 29122 Piacenza, Italy; andrea.minuti@unicatt.it (A.M.); erminio.trevisi@unicatt.it (E.T.)

**Keywords:** dairy calves, rumen development, forage, total mixed ration, rumination time

## Abstract

**Simple Summary:**

Preweaning nutrition plays a crucial role in the growth of dairy calves, determining their future productivity and overall health. Researchers are still studying the most appropriate dietary strategies in the preweaning and early post-weaning periods. In particular, the effect of different levels of forage inclusion in the diet of young cattle is not yet clear. Our study evaluated the effects of starter restriction and ad libitum inclusion of the total mixed ration on intake, growth performance, rumination time, and health status of Holstein dairy calves during preweaning and in the immediate post-weaning period. The results obtained from the present study indicate that providing TMR (high fiber content, with or without concentrate restriction) does not impair growth performance and health of calves, and at the same time it leads to a greater time spent for ruminating when calves are weaned. The latter indicates a better rumen development and likely a better rumen fermentation efficiency in post-weaning.

**Abstract:**

The aim of the current study was to evaluate the effect of the starter restriction and of the *ad libitum* TMR (total mixed ration) inclusion on intake, growth performance, rumination time (RT), and health condition of Holstein dairy calves during weaning. We randomly assigned thirty female Holstein calves (with an average weight of 38.5 ± 1.96 kg at birth) to one of three treatments. From 21 days of age, the calves were fed one of three treatments as follows: a control diet (CTR) with an *ad libitum* calf starter but without TMR; Treatment 1 diet (TRT1) with both an *ad libitum* calf starter and ad libitum TMR; Treatment 2 diet (TRT2) with *ad libitum* TMR and a restricted amount of a calf starter (50% of the intake recorder in the control group day by day). Calves in the TRT2 group, between 56 and 63 days of age, had a lower body weight (80.1; 79.5; 75.6 kg for the CTR, TRT1, and TRT2 groups, respectively) compared with CTR and TRT1 calves. This outcome is ascribed to the average daily gain (0.759; 0.913; 0.508 kg/day for the CTR, TRT1, and TRT2 groups, respectively), resulting also in TRT2 being lower than CTR or TRT1 calves. The inclusion of *ad libitum* TMR increased the rumination time, especially after weaning (15.28 min/h, 18.38 min/h, and 18.95 min/h for the CTR, TRT1, and TRT2 groups, respectively). Concerning the rumen metabolism and inflammometabolic response, overall, no differences were observed between the three dietary treatments. In conclusion, the results indicated that a TMR could partially replace a calf starter in weaning dairy calves, since neither growth performance nor health status were impaired. In addition, providing TMR (with or without concentrate restriction) led to a better rumen development and likely a better rumen fermentation efficiency in post-weaning.

## 1. Introduction

Feeding calves from birth to weaning is an important aspect of dairy farm economics as it is the first delicate step in obtaining a productive animal [1,2]. Even in this early period, promoting adequate morphological and physiological development of the rumen-reticular compartment leads to better performance both in young and in adult age [1].

It was well established that the volatile fatty acid production following the rumen fermentation of cereals stimulates the development of the rumen papillae [3]. However, ruminal fermentation of carbohydrates, especially starch, has also been shown to lower ruminal pH with negative repercussions on animal health and productivity [4]. Recently, some studies have instead reconsidered the positive role of the inclusion of forage in the diet of weaning calves. Many of these studies have incorporated forage into the diet, typically ranging from 5% to 25% of the total solid feed intake [4,5,6,7,8]. It has been found that the use of forage in the diet of young ruminants brings positive effects to the morphology of the rumen wall by increasing the thickness of the muscle layer and promoting the development of a healthy ruminal mucosa, while reducing plaque formation [8]. This effect was also observed when replacing 50% of barley or maize grain with maize silage in the diet of calves for 10 to 90 days [9].

In an experiment comparing a diet without corn silage and a diet supplemented with 15% corn silage, it was found that there was an improvement in dry matter intake (DMI), average daily gain (ADG), and body weight (BW), possibly due to the higher moisture content of corn silage, which helped to reduce dustiness and increase feed palatability [10]. However, other experiments on the inclusion of corn silage in the calf diet did not bring any advantages compared to the calf starter fed alone [11,12]. On the contrary, it was observed that feeding only corn silage slowed the growth of ruminal papillae and tended to compromise the intestinal morphology of calves [13], because the consumption of fibrous feed triggers the development of the rumen papillae [14]. Therefore, it is possible that corn silage can be used to partially replace the concentrate with few deleterious effects on calf growth and development. A recent study found that calves fed high-quality hay as the sole solid feed source had similar DMI, ADG, and post-weaning weights as calves fed a 70:30 mix of starter and forage [4].

Recently, in the attempt to evaluate the effects of including whole-maize silage in a calf starter ration or in a TMR on growth of blood metabolites and rumen fermentation, it was reported that including whole-corn silage in the TMR, but not in the calf starter ration, could represent a valid alternative to the diet for preweaning dairy calves [15]. Although all of these studies have demonstrated the benefits of including TMR in the diets of weaning calves, to date, there is still limited information on fiber sources and inclusion levels [16].

In summary, we hypothesized that providing *ad libitum* TMR and a restricted starter could encourage TMR intake and increase rumination time (RT) with consequent benefits for the performance of Holstein heifer calves during the pre- and post-weaning period. Hence, the objectives of the study were (1) to evaluate the effectiveness of the use of TMR in the calf’s diet during weaning on achieving an early and more effective ruminal activity than that recorded with a standard diet based only on milk and a starter, and (2) to observe whether a restriction of the starter could stimulate the intake of TMR.

## 2. Materials and Methods

### 2.1. Animal Management and Experimental Design

**Figure 1:**
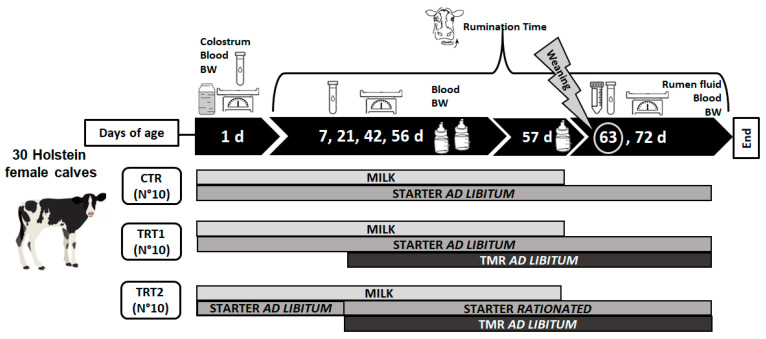
Experimental design of the study: sampling, measurements, and diets. Representation based on the days of life of the calves (d).

All performed procedures followed the common good clinical practices and received an institutional approval by the Animal Ethic Committee of the Magna Graecia University of Catanzaro (protocol number: 545/2020). The trial was carried out in a commercial dairy farm located in Bisignano (CS), Calabria (South Italy). A total of 30 heifer Holstein calves (BW: 38.5 ± 3.34 kg) born between December 2020 and January 2021 were enrolled in the trial as soon as after birth (Figure 1). Calves were separated from their dams within 2 h after birth and then moved to individual straw-bedded hutches (205 × 93 cm, providing an area of 5.04 m^2^) for the entire experimental period.

Calves were randomly assigned to 1 of 3 solid dietary treatments: (1) an *ad libitum* calf starter (CTR; n = 10); (2) an *ad libitum* calf starter plus, from 21 d of age, and *ad libitum* TMR (TRT1; n = 10); and (3) a restricted amount of a calf starter (50% of the intake recorded in the CTR group day by day) plus, from 21 days of age, and *ad libitum* TMR (TRT2; n = 10; Figure 1).

All calves were nipple-bottle-fed with 4 L of colostrum from the colostrum bank within 6 h from birth. If voluntary colostrum intake did not reach the required 4 L, the reaming colostrum quantity was administrated with an esophageal feeder. Colostrum of each calve was evaluated using a Brix refractometer (Dairy Tech, Incorporated, Weld County, Windsor, CO, USA) and through a FTIR analysis as suggested by Spina et al. [17].

A milk replacer (MR) (at a ratio of 150 g/L of water; 22.5% of crude protein (CP) and 19% of an ether extract (EE); ICIM spa, Mantova, Italy) was offered from the second meal to 56 days of age twice daily at 0630 and 1530 h: 4 L/d from 2 to 7 d, 5 L/d from 8 to 14 d, 6 L/d from 15 to 20 d, and 8 L/d from 21 to 42 d of age. Then, calves were stepped down to 6 L/d from 43 to 49 d and to 5 L/d from 50 to 56 d of age. From 57 to 63 d, calves were fed 2.5 L/d only once a day. All calves drank the entire amount of MR offered, with no significant exceptions among calves in the 3 groups.

At 63 d of age, calves were completely weaned and were fed only solid feeds according to the experimental protocol (Figure 1). Fresh water was offered daily *ad libitum.* Individual hutches were equipped with open buckets for water, starter, and TMR. The calf starter (BOVI-starter Pellet, PROGEO S.c.a., Reggio Emilia, Italy) and TMR were weighed and offered every morning at 0700 h, and refusals (i.e., the portion of starter and of TMR not consumed over a 24 h period) were weighed and registered daily. The commercial calf starter was formulated with the following ingredients (descending percentage): corn, soybean meal, soybean hulls, wheat bran, wheat middling, sugarcane molasses, corncob, sugar beet pulp, rice hull, calcium carbonate, calcium phosphate, soybean oil, and sodium chloride. The percentage of each ingredient is unknown as the calf starter was purchased from a licensed commercial feed mill. The TMR used in the experimental design was formulated according to the requirements of heifers between 6 and 12 months of life and was prepared daily on the farm. The ingredients of TMR (% DM) were as follows: grass silage (20.55) and corn silage (12.20), wheat straw (25.65), sunflower extract meal (12.32), cornmeal (9.12), and calf starter (20.17). Throughout the trial, samples of the calf starter and TMR were collected weekly for determination of nutritional composition according to Association of Official Analytical Chemists (AOAC, 2012) procedures: dry matter (DM), ether extract (EE), crude protein (CP), and ash. The neutral detergent fiber (NDF) content was determined through the method of Van Soest et al. [18]. The chemical composition and nutritional values of the calf starter and TMR are reported in Table 1.

### 2.2. Growth and Measurement of Rumination Behavior

Individual BW was recorded at 1, 7, 21, 42, 56, 63, and 72 d of age before morning feeding using an electronic scale. ADG was calculated as the difference between final BW and initial BW of each period, divided by the number of days on trial. Within 3 days of age, calves were fitted with the ear tag activity monitor (SCR eSense, Allflex, Irving, TX, USA). The Allflex eSense™ ear tag sensors were positioned in the middle of the calves’ left ears, following the manufacturer’s instructions. Furthermore, as demonstrated in previous studies, the system appears to be valid for accurate monitoring of daily rumination in cattle [19,20,21,22]. In short, rumination data were recorded in minutes per 1 h interval as an arbitrary number determined using a three-dimensional accelerometer that recorded the speed and angle of the ear movements. Data were transmitted from the ear tag every 20 min where the system software reported the rumination time using a series of internal algorithms, which are proprietary to Allflex [23,24]. Rumination time data (min/h) were processed to calculate rumination time over the entire trial and the average of rumination time in each of the following periods: 8 to 21 d, 22 to 42 d, 43 to 56 d, 57 to 63, and 64 to 72 days of age.

### 2.3. Blood Biomarkers and Analysis

Blood samples were collected through jugular venepuncture into heparinized tubes (BD Vacutainer; BD and Co., Franklin Lakes, NJ, USA) at 2, 7, 21, 42, 56, 63, and 72 days of life, in the morning before milk and starter distribution. After collection, blood samples were immediately cooled and then centrifuged at 3500× *g* for 16 min at 4 °C. Plasma was aliquoted and stored at −20 °C until a metabolite analysis. Samples of plasma were analyzed at 37 °C using an automated clinical analyzer (ILAB 650, Instrumentation Laboratory Company, Lexington, MA, USA) as described by Morittu et al. [25]. Commercial kits were used to measure glucose, urea, glutamic–oxaloacetic transaminase (GOT), total cholesterol, total protein, albumin, and total bilirubin (Instrumentation Laboratory SpA, Milan, Italy). Ceruloplasmin concentrations was measured using methods proposed by Bertoni et al. [26] adapted to ILAB 650 conditions, and myeloperoxidase (MPO, EC 1.11.2.2) was assessed as described by Bionaz et al. [27]. In addition, plasma samples collected at 1 day of life were used to measure serum total proteins (STPs) with an optical refractometer (HHTEC^®^, Kuehler Grund, Heidelberg, Germany)

### 2.4. Ruminal Fluid Samples and Analysis

Rumen fluid (50 mL) was collected at 63 and 72 d of age using a flexible esophageal tube (2 mm of wall thickness and 6 mm of internal diameter) and vacuum pump, 4 h after the morning feeding. Immediately, after discarding the first 2 mL of collected fluid to reduce potential saliva contamination, rumen pH was measured using a pH meter (pH 7+, Hanna Instruments, San Benedetto del Tronto, Italy). In addition, the samples were stored at −80 °C into 50 mL falcon tubes until the subsequent VFA analysis. Volatile fatty acids (VFAs) were quantified according to Minuti et al. [28] using a gas chromatograph (model 7820A GC, Agilent Technologies, Santa Clara, CA, USA) equipped with a DB-FFAP capillary column (30 m × 250 m × 0.25 m; Agilent J&W GC column) and a flame-ionization detector. Data about individual VFA were reported as molar proportions of the total VFA concentration.

### 2.5. Statistical Analysis

Data on BW, ADG, feed intake, blood, and rumen responses, as well as rumination activity, were analyzed using a linear mixed-effect model for repeated measures. We fixed the power (1 -β) to 80%, and we set the α-level at 0.05. The model included the effects of the diet “D” (CTR, TRT1, TRT2), time “T” (1, 7, 21, 42, 56, 63, 72 days of age of calves), and the interaction between diet and time “DxT” as fixed effects. The time was considered a repeated measure within the calf, and the calf was considered as a random effect. When not significant, the effect of the interaction was removed from the model. Normality of data distributions was assessed using Shapiro–Wilk and Kolmogorov–Smirnov tests. Tukey’s multiple comparison test was used to evaluate the difference between the averages. All results were expressed as expected marginal means (least squares means) ± SEM. Statistical difference was declared at *p* < 0.05 or considered as a trend at 0.05 < *p* < 0.10. Statistical processing was performed with Graph Pad PRISM software version 10.1.0 for Windows, La Jolla, CA, USA.

## 3. Results

### 3.1. Colostrum Quality

No differences in colostrum quality were found either using the optical refractometer (*p* = 0.79) or using the Milkoscan FT-MIR (*p* = 0.36). The average of colostrum Brix was 28.05, 28.45, and 27.70 for the CTR, TRT1, and TRT2 groups, respectively, whereas the average of total proteins of colostrum was 17.50, 17.19, and 16.59% for the CTR, TRT1, and TRT2 groups, respectively. Total serum protein content, analyzed with an optical refractometer, also revealed no differences between groups (*p* = 0.59). In fact, the average of total serum protein was 6.94, 7.12, and 7.20 g/dL at 2 days of life for the CTR, TRT1, and TRT2 groups, respectively.

### 3.2. Growth Performance and Feed Intake

**Figure 2:**
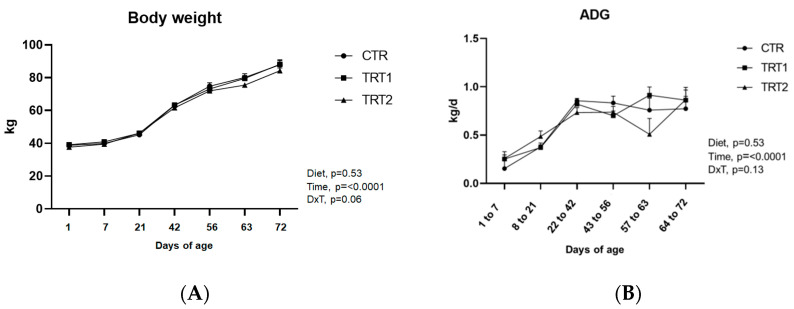
(**A**) Body weight at 1, 7, 21, 56, 63, and 72 days of age; (**B**) average daily gain (ADG) at 6 different periods of age. Error bars indicate the standard error of the mean (SEM).

Data on growth performance and feed intake are presented in Table 2. Overall, BW was not affected by the dietary treatments (*p* = 0.53), but increased with calf age (*p* < 0.01, Figure 2A). In the week before weaning, however, when the calves were between 56 (CTR = 74.80 ± 2.1 kg, TRT1= 73.10 ± 1.80 kg, TRT2 =71.9 ± 1.5) and 63 days old (CTR = 80.1 ± 2.2 kg, TRT1 = 79.5 ± 1.8 kg, TRT2 = 75.6 ± 1.2 kg), calves in the TRT2 group had a tendency (*p* = 0.09) to have lower BW than calves in the CTR group.

In post-weaning, groups did not differ for the BW (*p* = 0.18). Overall, ADG was not affected by the diet (*p* = 0.53) nor by the interaction (*p* = 0.13, Table 2, Figure 2B). However, as for BW, in the period between 56 and 63 days of age, a lower ADG was recorded for the TRT2 group (0.508 ± 0.164 kg/d) compared with the TRT1 group (0.913 ± 0.085 kg/d). The CTR group recorded intermediate ADG values (0.759 ± 0.124 kg/d).

**Figure 3:**
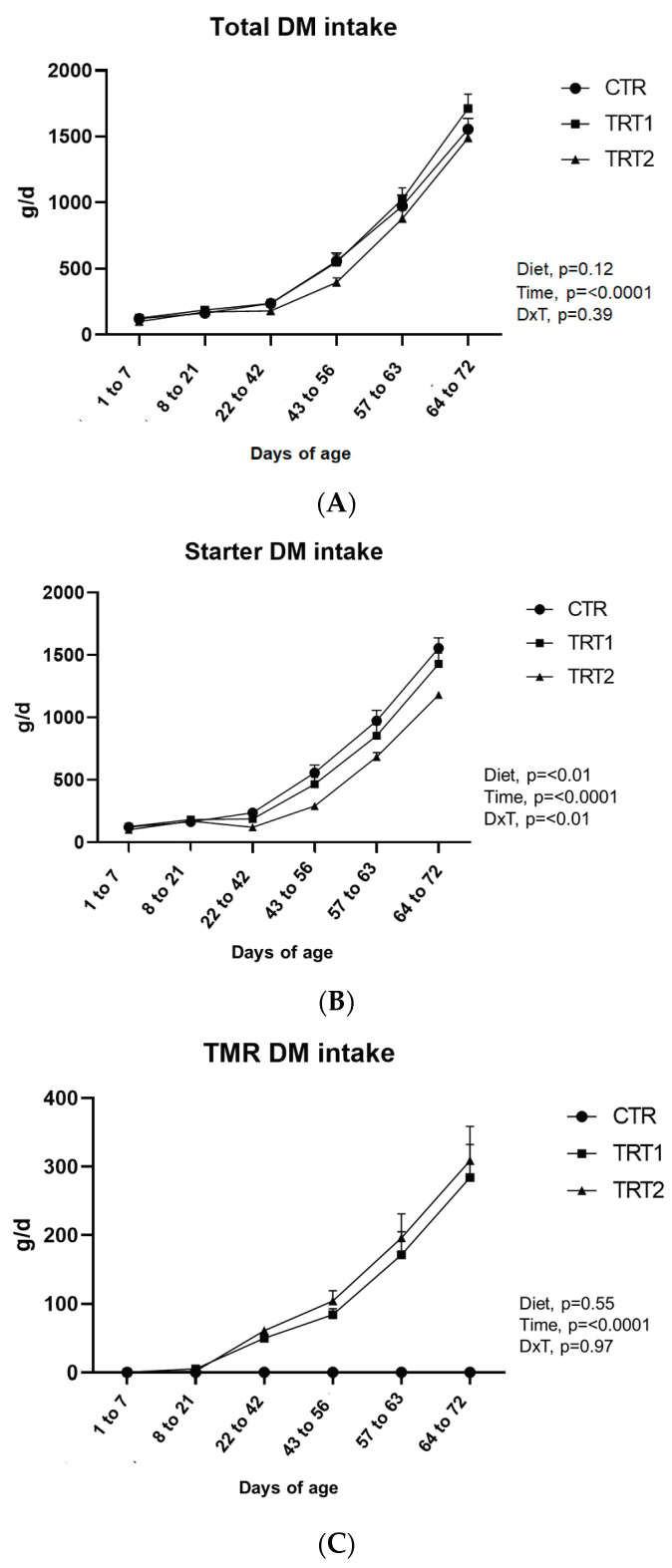
(**A**) The total dry matter feed intake from solid diet; (**B**) starter DM intake; (**C**) TMR intake; error bars indicate the standard error of the mean (SEM).

DMI increased with age (*p* < 0.0001). Overall, no differences were detected between groups (*p* = 0.12; Figure 3A). Since calves of TRT2 were fed with a restricted amount of the concentrate, starter intake was greater in CTR and TRT1 groups compared with the TRT2 group (*p* < 0.01; Figure 3B).

The interaction diet x time affected starter intake because from 22 to 72 days of life in the CTR and TRT1 groups, the consumption of the calf starter was greater than the TRT2 group (*p* < 0.01, Figure 3B).

The experimental protocol was set to offer calves of TRT2 approximately 50% of the starter intake recorded in the CTR group day by day. Thus, starter intake of TRT2 calves was on average 60% of that observed in the CTR group and 80% of that in the TRT1 group (achieving the planned purpose). Concerning the TMR on the DM basis, overall, the intake was not different between TRT1 and TRT2 groups (*p* = 0.57; Figure 3C) and increased over time (*p* < 0.0001), reaching nearly 200 g/d at weaning and 300 g/d in post-weaning. Total TMR consumption began to increase significantly starting from 56 days of age; in this period, in fact, the supply of milk was reduced in all groups.

Total energy intake differed according to the type of diet offered to the calves (*p* = 0.01); in particular, the energy intake of the CTR group (measured as UFL) was greater than the TRT2 group (*p* = 0.01), while the TRT1 group showed intermediate values, although a tendency to be higher was achieved compared with the TRT2 group (*p* = 0.06). According to the time, energy intake increased with the age (*p* < 0.0001), reaching about 1 UFL/head/day in the weaning period and approximately 1.7 UFL/head/day in the post-weaning period. The total NDF intake tended to be different among the three diets, and particularly the treated groups tended to have a higher NDF intake (*p* = 0.05) compared with the CTR calves. These differences were observed in two periods: the week before weaning and the 9 days after weaning. During these two periods, the two treated groups had an intake of approximately 100 g of NDF compared to the CTR group (D × T, *p* < 0.01).

Overall, the total starch intake was different among groups (*p* < 0.01); calves in the TRT2 group had less starch intake than the CTR (*p* < 0.01) and the TRT1 (*p* = 0.04) groups; meanwhile, we did not detect differences between the CTR and TRT1 groups (*p* = 0.62). Total CP and total ash intake did not vary between the groups, while total EE content was significantly lower in the TRT2 group (*p* = 0.04) than in the other two groups (Table 2).

### 3.3. Blood Parameters and Rumen Parameters

Mean values for blood metabolites of calves are presented in Table 3. All observed parameters were not influenced by the diet (*p* > 0.05), but only by the time (*p* < 0.01). Plasma urea, TP, and albumin values increased with age. Globulin gradually decreased from birth to weaning. The GOT, except the period from 7 to 21 days of age, in other observed periods significantly increased and remained stable between 15 and 17.60 U/L. CP increased from birth to 42 days of age and then remained constant at 1.42 (µmol/L). For MPO (U/L), stable values were observed throughout the preweaning period, while they increased significantly after weaning. There were no differences in the total protein content of serum observed between the three groups (*p* = 0.52).

Rumen fluid pH (Table 4) was not affected by the diet and time, but the interaction D × T tended toward significance (*p* = 0.07). After weaning, the TRT2 group tended to have a slightly lower pH than that observed before weaning, while in the other two groups, the rumen fluid pH remained stable.

Calves receiving TMR in their diets did not show a different VFA fermentation profile compared to the CON group (Table 4). The time effect was observed only for Isobutyrate (*p* = 0.03) and Isovalerate (*p* = 0.04) that were higher in preweaning respective to post-weaning (0.90 mmol/L vs. 0.72 mmol/L for Isobutyrate; 1.28 mmol/L vs 0.99 mmol/L for Isovalerate).

### 3.4. Rumination Time

**Figure 4:**
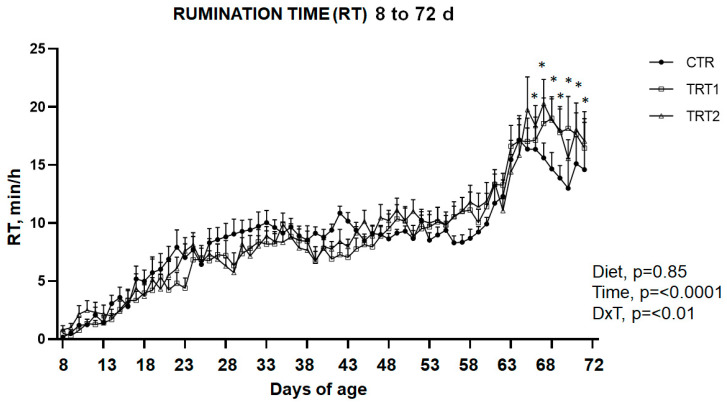
Daily rumination time (min/h) (mean ± SEM) for CTR, TRT1, and TRT2 groups between 8 and 72 days of age. * *p* < 0.05 (D × T interaction).

The application of the ear tags was well tolerated and did not cause any visible problems in the calves. Although ear tags were applied to the calves’ ears from day 2 of the trial, due to the time needed to set up the rumination monitoring system, we only considered data from 7 days of life. For the rumination time (RT, min/h), there was not a diet effect (*p* = 0.85), whereas it was found that there was a time effect (*p* < 0.0001) and an interaction D × T effect (*p* < 0.01). In detail, RT (min/h) increased with age during the monitored period, especially after weaning (Figure 4). On average, the RT for the whole observed period was 8.74 min/h for calves in the CTR group, 8.70 min/h for calves in the TRT1 group, and 9.12 min/h for calves in the TRT2 group. Interestingly, diet affected the RT only in the post-weaning period, from 3 days after weaning to the end of the trial (9 days after weaning). Indeed, in these days, calves in the CTR group showed a significantly lower RT than calves in the treated groups (*p* < 0.01; Figure 4). On days 3 and 4 after weaning, although calves in the TRT1 and TRT2 groups spent more minutes ruminating than calves in the CTR group, this difference reached significance only for the TRT2 group. Conversely, on the last day of observation (9 days after weaning), there was only a tendency (*p* = 0.07) for the treated groups to ruminate more than the control group. In the post-weaning phase, calves that did not receive TMR (CTR group) showed lower daily rumination activity than calves that did receive *ad libitum* TMR (15.28 min/h, 18.38 min/h, and 18.95 min/h for the CTR, TRT1, and TRT2 groups, respectively). As expected, the time spent ruminating was close to zero at the beginning of the trial and increased with increasing age. In general, in the period close to weaning and in the following one, the CTR group showed an increasingly lower RT compared to the TRT1 and TRT2 groups.

## 4. Discussion

The main objective of the present research was to investigate the effects of a complete TMR ration for healthy dairy calves, focusing on their growth performance, intake, and metabolic and ruminal characteristics. We mainly observed the immune status of all calves at the beginning of the trial. Values observed in the three groups exceeded the established limit values for plasma TP (>5.2 mg/dL) and plasma IgG (>1000.0 mg/dL) in Holstein calves, useful for monitoring failure of passive transfer (FPT) in dairy calves [29].

The effects of forage supply to young calves have been inconsistent and dependent on various factors, including forage source [30], amount of forage supplied [31], and chemical composition [6]. However, it seems that the optimal level of forage as a component of the ration is important for improving the growth performance and welfare status of dairy calves during the weaning period and after weaning. Our results indicated no differences in BW and ADG in calves fed only a starter *ad libitum* (CTR), a starter with *ad libitum* TMR (TRT1), or a restricted starter with *ad libitum* TMR (TRT2). The most critical period was the one around weaning, where calves of the TRT2 group showed the lowest BW (5 kg lower) compared to the other two groups. However, it is noteworthy to report that BW of TRT2 calves was in line with the BW range for Holstein calves at this age [32,33]. In the last observed period, the TRT1 and TRT2 groups showed the best ADG value (0.87 kg/d), which according to Shivley et al. [34], is an excellent daily gain (greater than 0.82 kg/day). Therefore, in this study, no calves had a “poor” ADG (ADG < 0.62 kg/day) indicated by Shivley et al. [34]. Various researchers have found few differences among calves fed only a calf starter compared with those fed forage mixtures. In a previous study where calves were fed pelleted or non-pelleted TMR, no significant effects on BW and ADG were found between the standard diet (starter only) and diets with TMR [35]. Kehoe et al. [13] investigated the effects of including corn silage in the initial feed provided to calves from birth until weaning and both BW and ADG did not differ from the control group. In general, the lack of an effect of the experimental protocol on DMI in the present trial, particularly in the TRT2 group, is also confirmed by the similar growth observed in all calves at the end of the trial. Calves in the TRT1 group, which received both a starter and *ad libitum* TMR, showed numerically the highest feed intake overall. In fact, this group consumed approximately 200 g more feed than the CTR and TRT2 groups. This indicates that the inclusion of forage did not affect starter intake, as documented by Hill et al. [36] in a trial on the addition of grass hay. In fact, as found by Castell et al. [37], the addition of forages in the diet of young calves creates a better environment for the rumen and it does not increase gut filling. These authors reported that providing free choice chopped forage to preweaning calves resulted in higher feed intake and better performance, without compromising digestibility. Regarding starter intake, the statistical difference that came up between the TRT2 group and the other two groups was predictable because the lower starter intake was planned with the experimental protocol. If we look at starter and TMR intake separately, we notice an interesting aspect that also explains the small difference in total DMI between the TRT1 and CTR group. In the TRT1 group, 60 g of the starter was replaced by the intake of TMR by calves. Costa et al. [38] suggested that ruminants can make feed choices from an early age and these choices are driven by nutritional demands, rumen function, or motivation to chew and ruminate. Webb et al. [39] also indicated that young calves preferred a diet with a low nutrient level but with a high level of chewing and rumination, although there was a high individual variability. Another explanation could lie in the higher moisture content of TMR compared with the starter, likely helping to reduce dustiness and increasing feed palatability [40]. The lower starter intake by the calves in the TRT2 group resulted in lower starch and EE content, and hence a lower energy intake (UFL). However, it is important to note that calves in the TRT2 group also had acceptable energy levels for dairy calves between 56 and 72 days of age, i.e., 0.90 UFL in preweaning and 1.50 UFL in post-weaning [34,41]. This outcome could explain the slightly lower growth tendency of the TRT2 group in the week before weaning.

In our study, we offered *ad libitum* TMR to the treated groups and it is known that TMR has a higher NDF content than a standard starter. In fact, NDF intake tended to be higher in the treated groups than in the CTR group. Studies on the use of various levels of fibrous sources in the diets of weaning calves led to the conclusion that the improvement in animal performance parameters depends on the total NDF content in the diet [36,37].

Suarez et al. [12] reported that calves consuming corn silage instead of a starter had higher ADG, higher protein digestibility, and increased final rumen mucosal weight.

In general, the recommended levels of NDF in the starter feed for dairy calves are between 15 and 25% [42] and the NDF content of the starter we used fell within this range, while the NDF content of the TMR was roughly 48%. Some researchers who fed young calves with diets at different levels of NDF did not observe behavioral abnormalities or worsened performance in animals receiving the diet with the higher than recommended levels of NDF [43]. Indeed, the provision of high-NDF forage was a good strategy to improve pelleted starter intake and performance of young suckling calves during weaning [44]. In the present study, diet did not compromise any blood parameters. In fact, some important parameters such as TP, albumin, and globulin presented concentrations similar to those reported in some previous works on preweaning calves [15,45]. In a recent study, regarding the inclusion of whole-plant corn silage in a starter or total mixed ration for preweaning dairy calves, where starch, EE, and NDF were lower in the experimental groups, authors did not find any changes in both performance and blood biomarkers [15].

The increase in rumen pH provides a more suitable environment for the growth of cellulolytic bacteria [46], which probably also plays a role in the digestibility of the fiber itself [47]. In our study, we did not observe wide differences in rumen pH between the three diets, with rumen pH above the threshold value applied for the diagnosis of SARA (sub-acute ruminal acidosis; threshold value pH ≤ 5.8; [48]). Offering only a calf starter would result in increased molar proportions of propionate and butyrate at the expense of acetate. Conversely, forage intake promotes cellulolytic microbial growth and results in an increase in the molar proportions of acetate in the rumen [49]. In our case, the molar concentrations of these VFAs did not vary between groups, although in the treated groups it was observed that there were slightly higher acetate and propionate concentrations together with lower butyrate concentrations. Our results agree with those of Suarez et al. [12], who found no change in total rumen VFA concentrations in TMR-fed dairy calves compared with the standard diet. EbnAli et al. [50] also found no significant differences in rumen acetate, propionate, and butyrate concentrations in calves fed TMR compared with standard diets during weaning. This aspect is probably due to the varying digestibility of NDF and of the starch [12,46].

The rumination activity in this study was evaluated through an innovative automatic monitoring system using ear sensors. As expected, the time spent ruminating was close to zero at the start of the trial and increased as the calves’ age and feed intake increased. In our study, the observed RT values of the 4 h/d period from 57 to 63 days of age and 7 h/d from 63 to 72 days of age are in line with the RT values observed by Lopreiato et al. [51] using Hr-Tag loggers in calves at the same age. In the study of Omontese et al. [24], an average RT of 9 h/d was reported in older calves. At the age of 10 weeks, rumination was well developed, similar to adult cows [51]. In fact, herein, all groups showed a daily RT above 340 min/d. Specifically, we recorded 454 min/d, 441 min/d, and 360 min/d for the TRT2, TRT1, and CTR groups, respectively. Another previous study defined a biologically acceptable RT equal to 297 ± 15 min/d for calves at 65 days of age and, in general, in the weaning period [52]. The average of rumination times estimated (over 24 h) by Rodrigues et al. [53] for calves in the preweaning, weaning, and post-weaning periods was 299 ± 19, 349 ± 29, and 457 ± 18 min/d, respectively. In the present study, calves that reached values of the latter study mentioned were the calves in the treated groups. Indeed, the time spent for ruminating by TRT1 and TRT2 groups was on average 240 min/d one week after weaning, 321 min/d at weaning, and 452 min/d after weaning. Focusing on the diet factor, our results showed that the TRT1 and TRT2 groups had a rumination time of 4 min/h more in the post-weaning period than the CTR group. Only from the third day after weaning was the RT greater in calves of TRT1 or TRT2 groups compared with calves in the CTR group.

The literature available to date indicates that the consumption of solid feeds and the amount consumed are the main factors in the initiation of rumen motility and thus rumination time [54]. In accordance with our results, van Ackeren et al. [55] found that calves fed a low-fiber TMR (26.2% NDF) spent less time chewing than calves fed a high-fiber TMR (31.3% NDF).

Previous studies have observed that RT is higher in calves fed grass and silage than in those fed only initial pelleted feed (approximately 5.5 vs. 4.2 h/d, respectively) at 7 weeks of age [56]. Porter et al. [5] also observed that calves fed with a pelleted starter alone started ruminating 2 weeks later than calves given forage. From these results, it was concluded that rumination stimulation associated with forage intake can also promote and accelerate rumen development, allowing calves to use forage effectively from an early age. Possible limitations to the present study include the low sample size and the use of straw as bedding. Recognizing these limitations is important to adequately contextualize the research and direct future studies to fully understand the impact of total mixed rations (TMRs) on calf development. Future studies could broaden observations of TMR use on the rumen microbiome and long-term development of heifers.

## 5. Conclusions

Overall, our results showed that feeding weaning Holstein calves with TMR ad libitum resulted in good growth levels without affecting health. Furthermore, TMR increased rumination, especially after weaning. Instead, the TRT2 group, which took 60% and 80% of the starter compared to the CTR and TRT1 groups, despite showing better rumination than the CTR group, showed less stable growth, which required greater monitoring during preweaning. We should note that the importance of TMR in weaning calves has above all the purpose of functioning as ruminal priming. In fact, it prepares the calves for the feed ration, composed exclusively of TMR, which they will receive from adult cows. Finally, TMR, used in part as a starter substitute, could also have positive economic implications on the total cost of the food ration of weaning calves, especially if the company prepares TMR itself.

## Figures and Tables

**Table 1:** Chemical composition and particle size distribution of Milk Replacer, calf starter, and TMR.

Chemical Composition	Milk Replacer ^1^	Starter ^2^	TMR
Dry Matter (DM) %	11.42	88.5	55.55
Crude Protein % of DM	22.45	19.21	17.38
Ether Extract % of DM	20.19	4.29	2.63
Ash % of DM	7.12	8.02	7.89
Starch % of DM	-	33.33	13.15
NDF % of DM	-	21.45	48.26
UFL ^3^/kg	1.10	1.05	0.73
Particle Size Distribution ^4^ %			
Long Particles	-	-	8.94
Medium Particles	-	-	39.32
Short Particles	-	-	14.09
Fine Particles	-	-	37.15

^1^ Contained Ca, 1.13%; P, 0.68%; Na, 0.57%; lysine, 2.15%. ^2^ Contained, for kg, 25,160 IU of vitamin A, 4390 IU of vitamin D3, 62.8 mg of vitamin E, 24 mg of vitamin B1, 1.6 mg of vitamin B2, 1.0 mg of vitamin B6, 0.0114 mg of vitamin B12, 8.0 mg of niacinamide, 16 mg of calcium iodide, 36.96 mg of manganese oxide, 38.63 mg of zinc oxide, and 0.5 mg of sodium. ^3^ Unité Fourragère Lait (Forage Unit for lactation) according to INRA 2007. ^4^ Particle size was determined using a Penn State particle separator, which separates the particles into 4 fractions on 4 sieves: long (>19 mm), medium (8–19 mm), short (4–8 mm), and fine (<4 mm).

**Table 2:** Effect of Diet (D) and Time (T) and interaction Diet × Time (D × T) on feed dry matter intake and growth performances in dairy calves from birth to 72 days of age.

	Diet				*p*-Value		
Item	CTR	TRT1	TRT2	SEM	D	T	D × T
BW (kg)	61.4	61.46	59.51	1.96	0.53	<0.0001	0.06
ADG (kg/d)	0.63	0.65	0.62	0.05	0.53	<0.0001	0.13
Total intake (g/d)	602	640	537	82.21	0.12	<0.0001	0.39
Starter intake (g/d)	602 ^a^	541 ^a^	425 ^b^	30.71	<0.01	<0.0001	<0.01
TMR intake (g/d)	-	99	112	6.50	0.55	<0.0001	0.97
Energy intake (UFL/d)	0.63 ^a^	0.64 ^a^	0.53 ^b^	0.05	0.04	<0.0001	0.28
Starch intake (g/d)	200 ^a^	192 ^a^	155 ^b^	27.41	0.01	<0.0001	0.03
CP intake (g/d)	116	120	101	9.13	0.11	<0.0001	0.46
NDF intake (g/d)	130	164	145	13.51	0.0522	<0.0001	<0.01
EE intake (g/d)	25.80 ^a^	25.62 ^a^	21.17 ^b^	3.74	0.04	<0.0001	0.22
Ash intake (g/d)	48.22	50.92	42.90	7.42	0.13	<0.0001	0.47

SEM = standard error of mean; values labeled with different lowercase letters are different for *p* < 0.05.

**Table 3:** Effect of diet (D) and days of age (T) and interaction Diet × Time (D × T) on plasma parameters of Holstein calves in weaning period.

	Diet				*p*-Value		
Item	CTR	TRT1	TRT2	SEM	D	T	D × T
Glucose (mmol/L)	5.36	5.94	5.73	0.21	0.34	<0.0001	0.75
Chol (mmol/L)	2.51	2.55	2.35	0.40	0.34	<0.0001	0.25
Urea (mmol/L)	4.75	4.52	4.41	0.35	0.44	<0.0001	0.14
TP (g/L)	62.11	63.02	62.71	2.25	0.82	<0.0001	0.20
Albumin (g/L)	29.59	29.18	29.05	1.36	0.74	<0.0001	0.22
Globulin (g/L)	32.52	33.9	33.73	2.01	0.50	<0.0001	0.34
GOT (U/L)	12.8	13.87	13.29	5.89	0.65	<0.0001	0.49
CP (µmol/L)	1.24	1.29	1.26	0.21	0.86	0.0003	0.89
MPO (U/L)	177.5	182.2	181.3	14.11	0.09	<0.0001	0.85

Chol = Cholesterol; TP = Total Protein; GOT = Glutamic Oxaloacetic Transaminase; CP = Ceruloplasmin; MPO = Myeloperoxidase. SEM = standard error of mean; mean values reported in the same column.

**Table 4:** Ruminal fermentation profile in dairy calves fed 3 diets (D), CTR, TRT1, TRT2, and observed at 2 times (T): Preweaning (63 days of age) and post-weaning (72 days of age).

	Diet				*p*-Value		
Item	CTR	TRT1	TRT2	SEM	D	T	D × T
Ruminal fluid pH	6.31	6.35	6.46	0.11	0.67	0.20	0.07
Urea (mmol/L)	7.79	8.73	6.93	1.77	0.62	0.75	0.73
D-Lactate (mmol/L)	131	134.8	146.2	43.04	0.96	0.62	0.79
L-Lactate (mmol/L)	143.7	146.4	156.4	51.62	0.96	0.68	0.79
Acetate (mmol/L)	45.25	46.76	47.66	5.92	0.91	0.78	0.46
Propionate (mmol/L)	21.46	23.47	24.69	4.95	0.69	0.45	0.54
Butyrate (mmol/L)	11.31	11.53	9.50	2.02	0.53	0.86	0.73
Isobutyrate (mmol/L)	0.62	0.92	0.86	0.16	0.12	0.03	0.06
Valerate (mmol/L)	3.07	3.05	2.61	0.88	0.74	0.12	0.41
Isovalerate (mmol/L)	0.95	1.33	1.14	0.24	0.32	0.04	0.20
Total VFA (mmol/L)	82.61	87.01	86.51	13.25	0.81	0.64	0.37

## Data Availability

Data supporting the findings of this study are available to anyone from the corresponding author upon reasonable request.
